# Cost and Utilization Trends of Lumbar Fusion

**DOI:** 10.1001/jamanetworkopen.2026.0452

**Published:** 2026-03-04

**Authors:** Brook I. Martin, Sohail K. Mirza, Brian A. Karamian, Andrew J. Schoenfeld, Hyunkyu Ko, Pradeep Suri, Darrel S. Brodke

**Affiliations:** 1 Department of Orthopaedics, University of Utah, Salt Lake City; 2 Thayer School of Engineering at Dartmouth College, Hanover, New Hampshire; 3 Inova Department of Orthopaedic Surgery, Fairfax, Virginia; 4 Department of Orthopaedic Surgery, Mass General Brigham, Harvard Medical School, Boston, Massachusetts; 5 Department of Rehabilitation Medicine, Clinical Learning, Evidence, and Research Center (CLEAR), University of Washington, Seattle

## Abstract

**Importance:**

The increasing cost of lumbar fusion has invited payment reforms, such as mandatory price limits by Medicare in 2026.

**Objective:**

To examine the cost, utilization, and procedural case-mix trends for different types of lumbar fusion from 2002 to 2023 in the United States.

**Design, Setting, and Participants:**

This cross-sectional analysis used survey-weighted data from the 2002 to 2023 National Inpatient Sample (NIS) and the 2016 to 2022 Nationwide Ambulatory Surgical Sample (NASS). From this nationally representative sample of inpatient and hospital-owned outpatient discharges, information on US adults aged 20 years and older undergoing lumbar fusion for any indication from January 2002 to December 2023 were included.

**Exposures:**

Lumbar fusion of any type (1-disc level or multilevel as well as single vertebral column or both anterior-posterior columns) with nonfusion surgery as a comparison.

**Main Outcomes and Measures:**

The main outcomes were the survey-weighted annual total of procedures, the mean age of patients undergoing lumbar fusion, the inflation-adjusted hospital costs, and the annual procedure rates per 100 000 population.

**Results:**

A total of 5 033 772 lumbar fusion admissions between 2002 and 2023 were included. In 2023, the cohort of patients undergoing 274 750 procedures had a mean (SD) age of 63.2 (12.9), with 142 815 (52.0%) female patients. Excluding 54 620 complex fusions, which were mostly multilevel anterior-posterior column fusions, there were 164 105 (50.1%) multilevel fusions, and 109 130 (51.3%) combined anterior-posterior column fusions. The age-adjusted population rate of inpatient fusion procedures increased from 60.1 (95% CI, 58.8-90.3) per 100 000 in 2002 (148 823 admissions) to a peak of 89.9 (95% CI, 89.6-90.3) in 2016 (284 180 admissions), before declining to 80.0 (95% CI, 79.7-80.4) by 2023 (273 235 admissions). Lumbar fusion performed in hospital-owned outpatient facilities was minimal in 2016 (6132 procedures, or 2.1% of total lumbar fusions) and 6.9 per 100 000 (27 331 procedures, or 9.8% of total lumbar fusions) in 2022. Adjusted inpatient hospital costs increased 265.3% from $3.86 (95% CI, $3.81-$3.92) billion in 2002 to $14.1 (95% CI, $13.9-$14.2) billion in 2023, and mean inpatient per-procedure cost increased from $25 849 (95% CI, $25 684-$26 015) in 2002 to $45 458 (95% CI, $45 207-$45 709) in 2023. Lumbar fusion primarily shifted from single column at 1 or 2 disc levels in 2002 (mean cost, $24 515; 95% CI, $24 361-$24 669) to multilevel anterior-posterior column fusion in 2023 (mean cost, $55 034; 95% CI, $54 420-$55 650).

**Conclusions and Relevance:**

In this cross-sectional study, lumbar fusion trends were marked by greater utilization of procedures overall, and especially involving multilevel and combined anterior-posterior column approaches and by greater use in the outpatient setting. Costs also increased at both the national and per-procedure levels.

## Key Points

**Question:**

What changes in lumbar fusion surgery utilization and costs occurred between 2002 and 2023?

**Findings:**

In this cross-sectional study of more than 5 million lumbar fusion admissions, adjusted inpatient hospital and mean inpatient per-procedure costs increased. There were increased trends toward fusions involving 2 or more disc levels and in anterior-posterior column fusion in more recent years as well as a trend toward procedures in outpatient facilities.

**Meaning:**

In this study, lumbar fusion trends were marked by greater utilization of multilevel and anterior-posterior approaches and greater use in the outpatient setting.

## Teaser

This cross-sectional study evaluates whether approaches to lumbar fusion surgery, utilization of the procedure, and costs have changed between 2002 and 2023 in the United States.

## Introduction

Spinal fusion surgery has invited increasing scrutiny because of widespread use, high costs, unexplained differences in surgical rates across geographic regions, and significant risk of complications. Furthermore, its effectiveness for certain common conditions has been called into question. The trend toward more fusions involving multiple intervertebral disc levels or both the anterior and posterior vertebral columns (AP fusion) has made spinal fusion a target for payment reforms, such as Medicare’s Transforming Episode Accountability Model (TEAM).^1^

While multilevel and AP fusion may improve spinal alignment and bone healing for specific pathology, small randomized clinical trials suggest they have higher early complication rates and similar patient-reported outcomes compared with single-level, single-column techniques for some common spine-related disorders.^2,3^ Nevertheless, a recent study showed greater reliance on fusion procedures irrespective of surgical indication (eg, disc herniation or stenosis without spondylolisthesis).^4^

We sought to describe recent trends in costs, utilization, and procedural case-mix for lumbar fusion in the United States. We hypothesized that the accelerated costs of lumbar fusion would be mainly attributed to the greater adoption of multilevel and AP fusions.

## Methods

### Study Design

Following the Strengthening the Reporting of Observational Studies in Epidemiology (STROBE) reporting guideline for cross-sectional studies, we performed an annual cross-sectional analysis of inpatient and hospital outpatient discharges available from the Agency for Healthcare Research and Quality’s Health Care Utilization Project (HCUP). Our study was exempted from institutional review board review and the requirement for informed consent by the University of Utah, which designated HCUP as public data.

### Data Sources

To assess population rates for each type of lumbar fusion performed in an inpatient setting, we analyzed the National Inpatient Sample (NIS)^5^ from January 2002 to December 2023, using age-specific population data from the US Census as the denominator.^6^ A 20% sample of all hospitals in the United States are included in NIS every year. All discharges from the sampled hospitals are included. The sampling strategy ensures that discharges are representative of hospitals designated as community hospitals in the American Hospital Association Annual Survey.^7^ Survey weighting and design variables requiring complex sampling statistics are included with NIS to ensure an unbiased national estimate.

To assess lumbar fusions performed in outpatient settings, we analyzed the Nationwide Ambulatory Surgical Sample (NASS)^8^ from 2016 to 2022. Outpatient data started including spinal fusion in 2016 when *International Statistical Classification of Diseases and Related Health Problems, Tenth Revision *(*ICD-10*) procedure codes were adopted. NASS only includes discharges from hospital-owned ambulatory surgery centers (less than 10% of total). As with NIS, NASS uses complex sampling methods to generate national estimates.

### Study Population

Adults aged 20 years or older undergoing inpatient lumbar, lumbosacral, and thoracolumbar fusion were selected using Medicare diagnosis related groups (DRGs) for spinal fusion (eTable 1 in Supplement 1). DRGs combine *ICD* diagnosis and procedure codes into groups defined by Medicare to set hospital payments. Each admission is coded by a single DRG. DRG codes for spinal fusion were revised in 2025 to separate 1-level from multilevel fusions. To characterize changes in multilevel and AP fusions, we applied the 2025 DRG definitions to data since 2016, when *ICD-10* procedure codes were adopted. Fusion operations combined with discectomy, laminectomy, interspinous spacer, or dynamic stabilizing device procedures are included. We excluded 2.4% of fusion operations because they were associated with DRGs not specific for fusion, such as “Soft tissue procedure with major complication or comorbidity” (DRG 500) and “Back and neck procedures except spinal fusion without (major) complication or comorbidity” (DRG 520).

Hospital outpatient department discharges in NASS are defined by up to 25 *Current Procedural Terminology* fields. We searched all these fields to identify spinal fusion and nonfusion (eg, decompressive laminectomy without fusion) procedures.

### Surgery Type

Using Medicare DRG nomenclature inpatient lumbar fusions were categorized as *noncervical spinal fusion*, *complex noncervical spinal fusion*, and *combined anterior-posterior spinal fusion*. Because the latter group included both cervical and lumbar fusions, we used *ICD-10* codes to restrict the cohort to only patients with lumbar diagnoses. We applied the 2025 revised DRG definitions to data from 2016 to 2023 to distinguish single-level from multilevel (2-7–level) fusions within single-column (either anterior or posterior) and within AP fusion techniques. Complex fusions are those involving 8 or more levels or fusions for fracture, infection, deformity, or cancer. For comparison, we also report trends in inpatient lumbar surgery not involving fusion.

### Covariates

Age group (5-year increments), sex, race, primary insurance payer, discharge disposition, and income quartile for the zip code of patient residence were included in the discharge registries. (However, NASS lacked race data until 2019, and NIS did not provide race in 2023.) To simplify reporting, we recoded race as Black, White, or additional groups (combining American Indian or Alaska Native, Asian, Hispanic ethnicity, Pacific Islander, and any other race and ethnicity not listed). Race and ethnicity are provided by HCUP partner organizations, and their reporting can vary by hospital. They are included to further characterize the sample. Primary payer was grouped as Medicare, Medicaid, private insurance, and other. The latter category (8.7% in NIS and 8.0% in NASS) included self-pay and charity or was unavailable. We estimated comorbidity using the enhanced version of the Charlson Comorbidity Index (CCI) from Quan et al^9^ based on all *ICD-10* diagnosis codes included on the claim. In addition, we identified claims with diagnosis codes for osteoporosis, osteoarthritis, or dementia because they are relevant to spinal fusion but not included in CCI.

### Surgical Indication

Each fusion case was classified by surgical indication using a validated algorithm that demonstrated high sensitivity and specificity for grouping back pain–related *ICD-10* codes into clinically distinct conditions (eTable 2 in the Supplement).^10^ The algorithm uses a hierarchy to group lumbar degenerative diagnoses into 5 categories: deformity (scoliosis or kyphosis), then spondylolisthesis, then spinal stenosis, then disc herniation, then disc degeneration. A patient with a diagnosis code for scoliosis is grouped into deformity even if he or she also has diagnosis codes for spondylolisthesis or spinal stenosis or disc herniation or disc degeneration associated with the procedure. Nondegenerative diagnosis codes for spinal fracture, infection, or cancer were classified as other. This classification allows distinction between well-established indications (eg, spondylolisthesis, scoliosis, fracture, infection, cancer)^11,12,13,14,15,16,17^ and more controversial indications (eg, spinal stenosis, disc degeneration, and disc herniation).^18,19,20,21,22,23,24^

### Hospital Costs

Costs of inpatient fusion admissions were summarized after multiplying the hospital charges of each NIS discharge to the corresponding All-Payer Inpatient Cost-to-Charge ratio, provided by HCUP.^5^ Excessively low (≤$100) or high (≥$10.0 million) charges are coded as missing by HCUP. Costs are distinct from hospital charges or actual reimbursement. They represent expenditures by a hospital to deliver a service, excluding professional fees, postacute care, and imaging. The Cost-to-Charge ratio was imputed at the group mean for a small number of hospitals for which it was missing. Costs in prior years of the analysis were inflated to their 2023 equivalents using the implicit price deflator.^25^ We did not report costs for hospital outpatient fusions because HCUP does not provide cost-to-charge ratios with NASS.

### Statistical Analysis

Cohort characteristics were described as survey-weighted frequency and percentages within variables (all categorical), with differences between the cohort in 2016 and 2023 reported as *P* values based on χ^2^ tests with a 2-sided α of .05. Building on prior epidemiology reports,^26,27^ we then estimated the population-based procedure rate of lumbar fusion and nonfusion procedures per 100 000 US residents, stratified by surgical approach, levels, indication, and setting (inpatient or outpatient). An age group–adjusted, survey-weighted Poisson regression was used to report trends in inpatient lumbar surgery rates from 2002 through 2023 and outpatient rates from 2016 to 2022. We added linear splines with knots set in 2005, when the DRG for complex fusion started, and in 2016, when DRGs distinguished between 1-level and multilevel fusion procedures. By incorporating the log of the US Census annual population (denominator) as an offset, we estimated rates per 100 000, separately stratified by multilevel, combined AP fusion, and indication.

A survey-weighted generalized linear regression (gamma family distribution with log link) with the same splines, adjusting for year of surgery, age (5-year increments), sex, CCI, osteoporosis, osteoarthritis, and dementia, was used to estimate mean and total annual inpatient hospital costs from 2016 to 2023. Estimates were stratified by multilevel, combined AP fusion, and indications. All analysis was performed using Stata/MP version 18.5 (StataCorp).

## Results

### Cohort Characteristics

A total of 5 033 772 admissions for lumbar fusion surgery 2002 and 2023 were included. In 2023, the final year of our study, the cohort of patients undergoing 274 750 procedures had a mean (SD) age of 63.4 (12.9), with 142 815 (52.0%) female patients (Table). Excluding 54 620 complex fusions, which were mostly multilevel AP fusions, there were 164 105 (50.1%) multilevel fusions and 109 130 (51.3%) AP fusions. Using the 2025 DRG definitions, survey-weighted US admissions of inpatient lumbar fusion increased from 148 823 procedures in 2002 to 273 235 procedures in 2023. During the study period, inpatient fusion shifted toward older patients, increasingly covered by Medicare and with greater comorbidity. The volume of lumbar fusions increased 108% among patients aged 65 years or older from 25.4% of total fusions in 2002 to 52.8% in 2023 (eFigure in Supplement 1).

**Table.** Descriptive Characteristics of Inpatient Lumbar Fusion Procedures, 2002 to 2023, Based on National Inpatient Sample

Characteristic	Procedures, No. (%)^a^		*P* value
	2002 (n = 148 823)	2023 (n = 273 235)	
Sex			
Male	63 537 (45.0)	131 935 (48.0)	<.001
Female	77 579 (55.0)	142 815 (52.0)	
Age group, y			
20-24	2221 (1.6)	2260 (0.8)	<.001
25-29	4006 (2.8)	2650 (1.0)	
30-34	8359 (5.9)	4295 (1.6)	
35-39	13 619 (9.7)	7005 (2.5)	
40-44	16 965 (12.0)	10 245 (3.7)	
45-49	17 282 (12.2)	13 565 (4.9)	
50-54	15 753 (11.1)	21 455 (7.8)	
55-59	14 765 (10.5)	29 070 (10.6)	
60-64	12 327 (8.7)	38 995 (14.2)	
65-69	11 177 (7.9)	47 765 (17.4)	
70-74	10 980 (7.8)	45 500 (16.6)	
75-79	8721 (6.2)	34 590 (12.2)	
80-84	3893 (2.8)	13 665 (5.0)	
≥85	1050 (0.7)	3695 (1.3)	
Race^b^			
White	78 178 (83.4)	201 500 (79.9)	<.001
Black	6018 (6.4)	21 400 (8.5)	
Additional groups^c^	9533 (10.2)	29 380 (11.6)	
Primary payer			
Medicare	39 459 (28.0)	144 370 (52.7)	<.001
Medicaid	5616 (4.0)	19 845 (7.2)	
Commercial	67 527 (47.9)	89 110 (32.6)	
Other	28 411 (20.1)	20 435 (7.5)	
Zip code income quartile^d^			
First (low income)	34 833 (21.1)	62 275 (23.0)	<.001
Second	42 646 (25.9)	73 215 (27.0)	
Third	47 097 (28.6)	71 185 (26.3)	
Fourth (high income)	40 277 (24.4)	64 295 (23.7)	
Discharge disposition			
Routine	101 035 (71.9)	160 935 (58.6)	<.001
Transfer to short-term hospital	556 (0.4)	1075 (0.4)	
Transfer to skilled nursing, intensive care, or other	23 153 (16.5)	53 810 (19.6)	
Home health care	15 301 (10.9)	57 665 (21.0)	
Against medical advice, died, or unknown	381 (0.3)	1250 (0.5)	
Spine diagnosis			
Axial	29 135 (20.6)	7610 (2.8)	<.001
Disc herniated	35 832 (25.4)	7895 (2.9)	
Stenosis	17 068 (12.1)	81 125 (29.5)	
Spondylolisthesis	41 195 (29.2)	115 160 (41.9)	
Scoliosis	7886 (5.6)	46 825 (17.0)	
Other nondegenerative (eg, fracture)	10 000 (7.9)	16 145 (5.9)	
Charlson Comorbidity Index			
0	102 629 (72.7)	128 555 (46.8)	<.001
1	28 025 (19.9)	68 290 (24.9)	
≥2	10 462 (7.4)	77 915 (28.4)	
Osteoporosis			
No	137 221 (97.2)	259 605 (94.5)	<.001
Yes	3895 (2.8)	15 155 (5.5)	
Osteoarthritis			
No	135 151 (95.8)	246 045 (89.5)	<.001
Yes	5964 (4.2)	28 715 (10.5)	
Dementia			
No	148 489 (96.8)	261 805 (95.3)	<.001
Yes	333 (3.2)	12 955 (4.7)	

^a^ Frequencies based on weighted sample applying complex sampling statistics. Total frequencies are not always equal across characteristics due to some missingness and/or estimation rounding. Percentages without characteristics do not always amount to 100% due to rounding.

^b^ Race data were not available in 2023; reported values are for 2022.

^c^ Additional groups includes American Indian or Alaska Native, Asian, Hispanic, and Pacific Islander individuals as well as those identifying as any race or ethnicity not listed.

^d^ Income information reported for 2005, when it first became available.

### Fusion Rates

**Figure 1.**
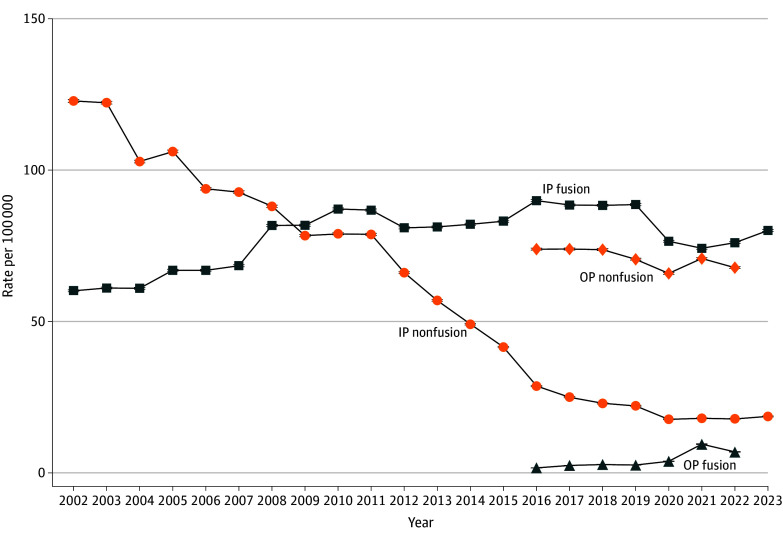
Frequency Graph of US Trends in Rates of Lumbar Fusion and Nonfusion Procedures Annual US population rate of inpatient (IP; 2001-2023) and hospital-owned outpatient (OP; 2016-2022) lumbar fusion and nonfusion spinal procedures; 95% CIs are present but are nearly imperceptible due to their narrow range.

The adjusted rate of inpatient lumbar fusion increased from 60.1 (95% CI, 58.8-90.3) per 100 000 in the US population in 2002 (148 823 admissions) to 89.9 (95% CI, 89.6-90.3) per 100 000 in 2016 (284 180 admissions) and then declined to 80.0 (95% CI, 79.7-80.4) per 100 000 by 2023 (Figure 1). The decrease in 2020 may have been related to COVID-19 restrictions on elective surgery^28^ as well as a shift toward outpatient settings. Lumbar fusions in hospital-owned ambulatory surgery centers increased 345.7% from 2016 (6132 procedures, or 2.1% of total lumbar fusions) to 2022 (27 331 cases, or 9.8% of total lumbar fusions), contributing 6.9 per 100 000 procedures in 2022 (eTable 3 in Supplement 1).

### Hospital Costs

**Figure 2.**
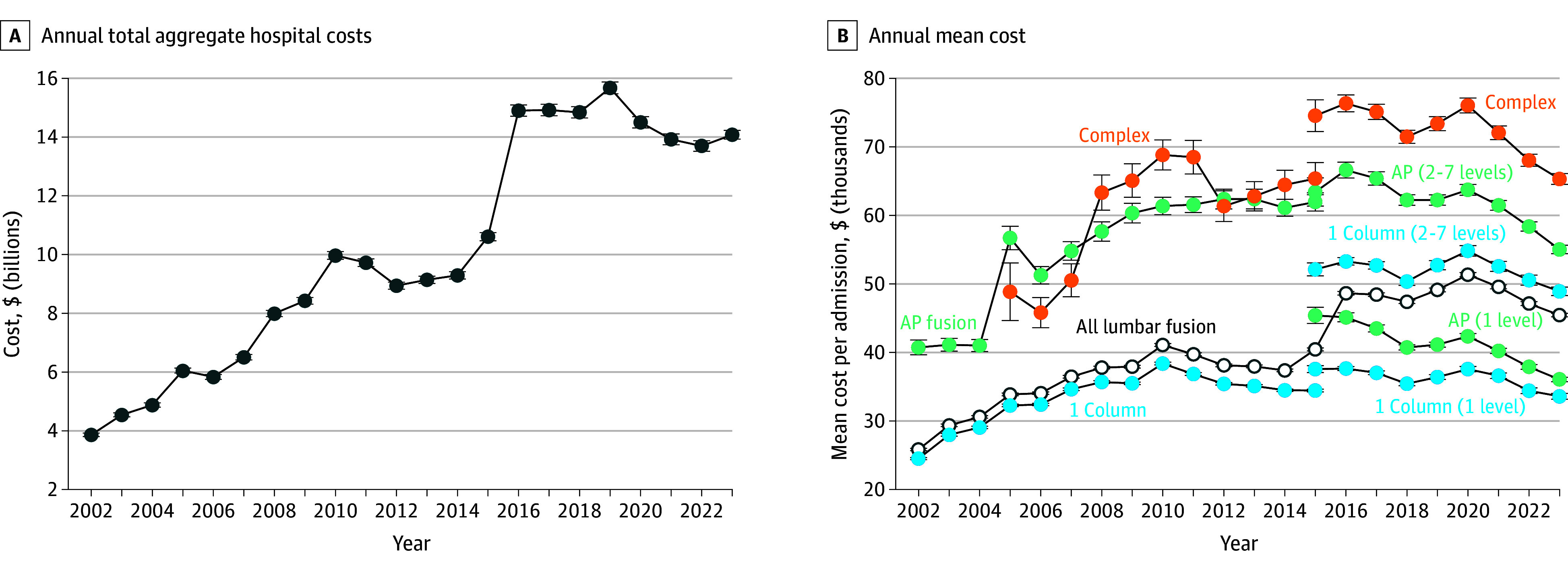
Frequency Graphs of US Trends in Aggregate and Mean Costs of Inpatient Lumbar Fusion Procedures A, Annual total aggregate hospital costs for inpatient lumbar fusion in the United States. B, Annual mean cost per lumbar fusion case in the United States, overall and by Diagnosis Related Group. We applied 2025 revised Diagnosis Related Group definitions to data starting in 2016 to distinguish 1-level and multilevel (2-7–disc levels) procedures. Based on National Inpatient Sample, 2002-2023. All costs are inflation-adjusted, with earlier years expressed in 2023 US dollar equivalents; 95% CIs are present but are nearly imperceptible due to their narrow range. AP indicates combined anterior-posterior column.

Inflation-adjusted total annual cost of inpatient lumbar fusion increased 265.3% from $3.86 (95% CI, $3.81-$3.92) billion in 2002 to $14.1 (95% CI, $13.9-$14.2) billion in 2023 (Figure 2A). Mean adjusted cost of inpatient lumbar fusion increased 75.9%, from $25 849 (95% CI, $25 684-$26 015) in 2002 to $45 458 (95% CI, $45 207-$45 709) in 2023 (Figure 2B; eTable 4 in Supplement 1).

The mean adjusted inpatient cost in 2023 for a 1-level single-column fusion was $33 610 (95% CI, $33 178-$34 042). A 1-level AP fusion was $36 071 (95% CI, $35 743-$36 400). A multilevel single-column fusion was $48 931 (95% CI, $48 315-$49 547). Excluding uncommon complex fusions that are not used for common degenerative disease, the cost of a multilevel AP fusion was $55 034 (95% CI, $54 420-$55 650) (Figure 2B; eTable 5 in Supplement 1).

### Fusion Types

**Figure 3.**
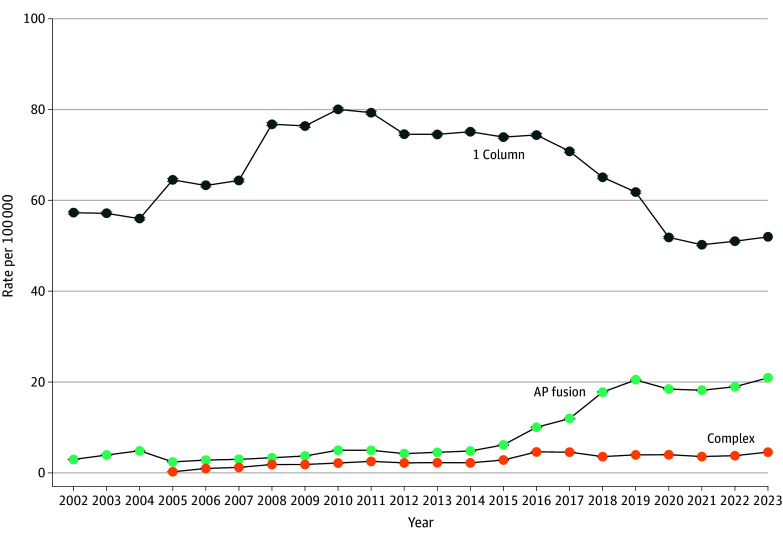
Frequency Graphs of US Trends in Rates of Inpatient Lumbar Fusion, by Fusion Type Annual US population rate per 100 000 of lumbar fusion, by type based on Diagnosis Related Group; 95% CIs are present but are nearly imperceptible due to their narrow range. AP indicates combined anterior-posterior column.

The rate of multilevel AP fusion increased since 2017, with a commensurate decline in 1-level single-column fusion (Figure 3; eTable 6 in Supplement 1). Excluding complex fusion, AP fusions increased as a percentage of the total fusion rate from 19.6% (55 695 of 281 470 procedures) in 2016 to 41.1% in 2023 (112 170 of 273 235 procedures). Multilevel fusions increased as a percentage of the total fusion rate from 44.8% in 2016 to 50.1% in 2023, with 49.5% of all multilevel fusions involving an AP approach (increasing from 23.3% in 2016). As a percentage of the total rate, 1-level single-column fusions declined from 43.7% in 2016 to 25.1% in 2023. As a percentage of the total fusion rate, complex fusion (involving ≥8 levels, fracture, infection, or cancer) increased from 11.3% in 2016 to 19.0% in 2023.

### Fusion Indications

**Figure 4.**
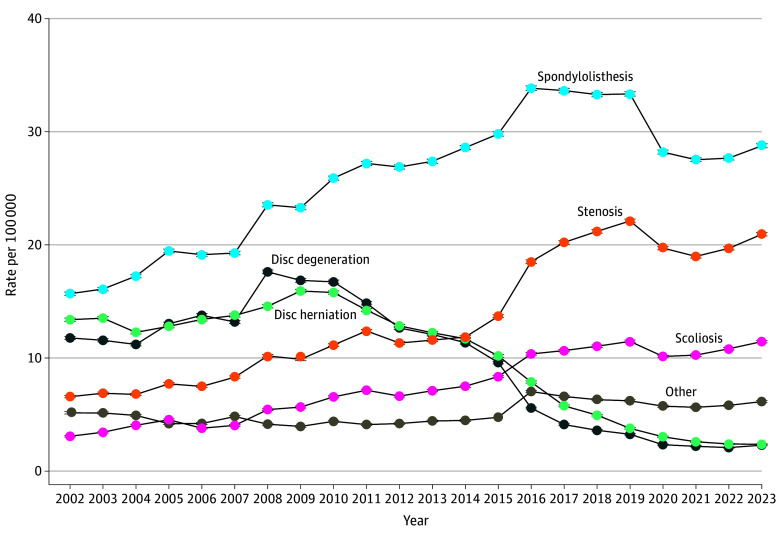
Frequency Graph of US Trends in Rates of Inpatient Lumbar Fusion, by Indication Annual US population rate per 100 000 of lumbar fusion, by hierarchical diagnosis at time of procedure; 95% CIs are present but are nearly imperceptible due to their narrow range.

From 2002 to 2023, the rate of fusion procedures for adult scoliosis increased 271.2% (319.1% by volume), spinal stenosis increased 218.0% (375.6% by volume), and spondylolisthesis increased 83.4% (177.1% by volume). There were commensurate decreases of 80.5% in the rate for disc degeneration (−73.6% by volume) and 82.3% decrease in fusion for disc herniation (−77.9% by volume) (Figure 4).

## Discussion

Annual hospital costs for inpatient lumbar fusion increased 265.3% from 2002 to 2023, not including costs for the 6.9% of fusion procedures performed in hospital-owned outpatient settings, for which cost data were not available. The increase appeared to be driven by changes in procedural case-mix for lumbar fusion, including an 820% increase in the rate of AP fusion since 2002, and a 1303.1% increase in the rate of complex fusion since 2006. Multilevel fusion accounted for nearly half of all fusion-based procedures in 2023 (48.6% of total). The adoption of these types of fusion from 2002 to 2023 outpaced the increase in total volume (88.3% increase), population rate (19.1% increase), and an aging population (108% increase by volume among those age ≥65 years).

Fusion is essential to restore structural stability and prevent neurologic injury in patients with unstable fractures from trauma,^11^ pathological fractures from cancer,^12,13^ instability from infection,^14,15^ and progressive deformity from scoliosis.^16,17^ Surgical treatment options for these indications depends on the degree of anatomic disruption caused by the condition. Clinical studies indicate that lumbar fusion for degenerative spondylolisthesis, the most common indication for lumbar fusion in our analysis, results in better patient-reported outcomes (PROs) than nonsurgical treatment at 2, 4, and 8 years of follow-up.^29,30,31^ However, complication rates are higher with multilevel and AP fusion.^32^ Fusion for other degenerative indications (ie, disc degeneration, disc herniation, and spinal stenosis without spondylolisthesis) remains controversial,^33,34,35,36^ yet these indications accounted for the plurality of total lumbar fusions in 2023.

The increase in fusion for scoliosis may reflect improvements in techniques and instrumentation that facilitate safer surgery. Multilevel and AP fusion for adult deformity have high rates of complications but the literature is conflicted regarding improvement in PROs.^37,38,39^ Critical adverse events are rare but can be catastrophic. Higher complication risks may be acceptable at times, for example with patients facing paralysis and progressive neurological deficit, but they may not be acceptable for patients with chronic low back pain if it remains unclear whether there is a benefit in functional outcome or pain.

Our findings suggest that decompression procedures are largely being replaced by fusion. Hospital outpatient nonfusion lumbar surgery did not increase proportional to the decline in inpatient nonfusion procedures. This suggests that nonfusion cases, such as decompressive laminectomy, have been replaced by fusion procedures more than they have shifted to outpatient settings. Lumbar fusion added to decompression (laminectomy and foraminotomy) for disc herniation or spinal stenosis without spondylolisthesis remains controversial.^22,23,34^ Since patients with leg symptoms (leg pain, numbness, weakness, neurogenic claudication, or radiculopathy) frequently also have associated back pain, and since their radiographs and magnetic resonance imaging usually show anatomic abnormalities at multiple levels, some surgeons believe that the use of multilevel and AP fusion to address these abnormalities and restore spinal alignment may reduce back pain.^40,41,42^

Another factor influencing increased adoption of multilevel and AP fusion is the proliferation of enabling surgical technologies. Multilevel AP fusions in the past were performed only by a few surgeons practicing at major academic centers. Now, recent graduates of spine fellowships are trained in complex surgical procedures using image guidance,^43^ surgical navigation,^44^ and robotic assistance.^45,46^ Today, even smaller hospitals acquire the necessary equipment to allow surgeons to perform more complex surgery.^47^

Starting on January 1, 2026, Medicare’s TEAM will replace current fee-for-service payments with a mandatory 30-day bundled payment model at all hospitals within randomly selected US regions. Price targets for each type of fusion will be set according to regional averages. Understanding the influence of procedural case mix on hospital financial risk will become a priority. TEAM is intended to mitigate “provider inducement,” wherein surgeons elect to use more expensive procedures because of a financial incentive.^48^

### Limitations

This study has limitations. DRGs for fusion capture a heterogeneous population of patients and fusion procedures, varying by indication, symptomatology, disability, and comorbidity. Reliance on coding algorithms may lead to some misclassification of cases by fusion procedure or indication. Although validated for classifying spine surgery, *ICD* diagnosis and procedure codes lack clinical detail, such as severity of pain, functional outcomes, or neurological symptoms, and do not fully capture surgical complexity. NIS and NASS data do not facilitate reporting of readmission, postdischarge complications, or PROs, and thus, no inference of procedural appropriateness can be made. Surgeons may be opting for multilevel or combined AP fusions based on the belief that they provide better vertebral stability, reduce readmission, and improve outcomes.

## Conclusions

In this annual cross-sectional study of nationally representative inpatient and hospital outpatient discharge registries, lumbar spine surgery evolved toward greater utilization of fusion, more multilevel and AP fusions, and increased use of outpatient fusion. Fusions for spinal stenosis also appeared to be on the rise. The clinical advantages of multilevel and AP fusion procedures need to be further investigated, specifically examining readmission, complications, and PROs. The proclivity to use such interventions may derive from optimizing techniques to allow for safer surgery in patients previously deemed unsuitable for them, enabling technologies, more broadly adopted and accepted surgical techniques, and the desire to leverage outpatient interventions. Payment reform efforts such as TEAM, which are designed to incentivize less expensive procedures that have lower risks of postsurgical adverse events and reduced need for perioperative health care utilization, will challenge these trends.

## Notes

Supplement 1. **eTable 1.** Spine-Related Diagnosis Related Groups **eTable 2.** Hierarchical Diagnosis Codes **eTable 3.** Annual Volume of Hospital-Owned Outpatient Departments Lumbar Surgery (Fusion and Nonfusion) Among Patients Aged 20 Years or Older, by *Current Procedural Terminology* Codes **eTable 4.** Annual Inflation Adjusted Mean Cost Per Case and Total Hospital Costs (ie, “National Bill”) for Inpatient Lumbar Fusion, 2002-2023 **eTable 5.** Annual Trends in Mean Inpatient Hospital Cost for Lumbar Fusion and Nonfusion Operations, by Diagnosis Related Groups (2002-2015) and 2025 Revised Diagnosis Related Groups That Separate 1-Level and Multilevel Procedures (2016-2023) **eTable 6.** Annual Trends in Rates (per 100 000) of Inpatient Discharges for Lumbar Fusion, by Diagnosis Related Groups (2002-2015) and 2025 Revised Diagnosis Related Groups That Separate 1-Level and Multilevel Procedures (2016-2023) **eFigure.** Age-Specific Rate (per 100 000) and Volume (in Thousands) of Inpatient Lumbar Fusion in the United States for 2002 and 2023

Supplement 2.
Data Sharing Statement
