# Supplementary material for: Cost and Utilization Trends of Lumbar Fusion
Source: JAMA Netw Open. 2026 Mar 4;9(3):e260452. doi: 10.1001/jamanetworkopen.2026.0452 (PMC12961518; doi:10.1001/jamanetworkopen.2026.0452)
Supplement: Supplement 1. — eTable 1. Spine-Related Diagnosis Related Groups eTable 2. Hierarchical Diagnosis Codes eTable 3. Annual Volume of Hospital-Owned Outpatient Departments Lumbar Surgery (Fusion and Nonfusion) Among Patients Aged 20 Years or Older, by Current Procedural Terminology Codes eTable 4. Annual Inflation Adjusted Mean Cost Per Case and Total Hospital Costs (ie, “National Bill”) for Inpatient Lumbar Fusion, 2002-2023 eTable 5. Annual Trends in Mean Inpatient Hospital Cost for Lumbar Fusion and Nonfusion Operations, by Diagnosis Related Groups (2002-2015) and 2025 Revised Diagnosis Related Groups That Separate 1-Level and Multilevel Procedures (2016-2023) eTable 6. Annual Trends in Rates (per 100 000) of Inpatient Discharges for Lumbar Fusion, by Diagnosis Related Groups (2002-2015) and 2025 Revised Diagnosis Related Groups That Separate 1-Level and Multilevel Procedures (2016-2023) eFigure. Age-Specific Rate (per 100 000) and Volume (in Thousands) of Inpatient Lumbar Fusion in the United States for 2002 and 2023 [file jamanetwopen-e260452-s001.pdf]

Supplemental Online Content

Martin BI, Mirza SK, Karamian B, et al. Cost and utilization trends of lumbar fusion. *JAMA Netw Open.* 2026;9(3):e260452. doi:10.1001/jamanetworkopen.2026.0452

eTable 1. Spine-Related Diagnosis Related Groups

eTable 2. Hierarchical Diagnosis Codes

eTable 3. Annual Volume of Hospital-Owned Outpatient Departments Lumbar Surgery (Fusion and Nonfusion) Among Patients Aged 20 Years or Older, by *Current Procedural Terminology* Codes

eTable 4. Annual Inflation Adjusted Mean Cost Per Case and Total Hospital Costs (ie, “National Bill”) for Inpatient Lumbar Fusion, 2002-2023

eTable 5. Annual Trends in Mean Inpatient Hospital Cost for Lumbar Fusion and Nonfusion Operations, by Diagnosis Related Groups (2002-2015) and 2025 Revised Diagnosis Related Groups That Separate 1-Level and Multilevel Procedures (2016-2023)

eTable 6. Annual Trends in Rates (per 100 000) of Inpatient Discharges for Lumbar Fusion, by Diagnosis Related Groups (2002-2015) and 2025 Revised Diagnosis Related Groups That Separate 1-Level and Multilevel Procedures (2016-2023)

eFigure. Age-Specific Rate (per 100 000) and Volume (in Thousands) of Inpatient Lumbar Fusion in the United States for 2002 and 2023

This supplemental material has been provided by the authors to give readers additional information about their work.

eTable 1. Spine-Related Diagnosis Related Groups

Diagnosis Related Group (DRG) codes for lumbar fusion, 2002-2022. Prior to the 2025 DRG changes for fusion, the combined anterior and posterior fusion DRG included both cervical and non-cervical spinal fusion. To restrict our analysis to only non-cervical spinal fusion, we required these DRG's to be co-coded with a thoracolumbar, lumbar or lumbosacral diagnosis code on the admission claim. Complex fusion those involving 8+ disc levels, vertebral fracture, infection, deformity or cancer.

<table>
<tr><th>Year</th><th colspan="2">Combined anterior posterior spinal fusion</th><th>Complex non-cervical spinal fusion</th><th colspan="2">Non Cervical Spinal fusion</th></tr>
<tr><td>2002 – 10/2007</td><td colspan="2">496 - Combined anterior/posterior spinal fusion;</td><td><u>Starting 10/2005:</u>
546 - Spinal fusion except cervical with curvature of the spine or malignancy.
546 - Spinal fusion except cervical with curvature of the spine or malignancy.</td><td colspan="2">497 - Spinal fusion except cervical w/ complication or comorbidity.
498 - Spinal fusion except cervical without complication or comorbidity.</td></tr>
<tr><td>10/2007 - 2024</td><td colspan="2">453 - Combined anterior/posterior spinal fusion w major complication or comorbidity.
454 - Combined anterior/posterior spinal fusion w complication or comorbidity.
455 - Combined anterior/posterior spinal fusion w/o complication or comorbidity or major complication or comorbidity.</td><td>456 - Spinal fusion except cervical with spinal curvature/malignancy/infection or 9+ vertebrae fused with Major complication or comorbidity.
457 - Spinal fusion except cervical w spinal curvature/malignancy/infection or 9+ vertebrae fused with complications or comorbidity.
458 - Spinal fusion except cervical with spinal curvature/malignancy/infection or 9+ vertebrae fuses without complications or comorbidity or major complications or comorbidity.</td><td colspan="2">459 - Spinal fusion except cervical w major complication or comorbidity.
460 - Spinal fusion except cervical without major complication or comorbidity.</td></tr>
<tr><td rowspan="2">2025 revised DRGs specific to fusion (applied to data after 2016 in analysis)</td><td>Lumbar 1-level AP</td><td>Lumbar AP Multilevel</td><td>Complex lumbar fusion (curvature, malignancy, infection, 7+ levels)</td><td>Lumbar 1-level 1-column</td><td>Lumbar 1-column, multilevel</td></tr>
<tr><td>402 - Single level combined anterior and posterior spinal fusion except cervical.</td><td>426 - Multiple level combined anterior and posterior spinal fusion except cervical with major complication or comorbidity or custom-made anatomically designed interbody fusion device.
427 - Multiple level combined anterior and posterior spinal fusion except cervical with complication or comorbidity.
428 - Multiple level combined anterior and posterior spinal fusion except cervical without complication or comorbidity or major complication or comorbidity.</td><td>456 - Spinal fusion except cervical with spinal curvature, malignancy, infection or extensive fusion with major complication or comorbidity.
457 -Spinal fusion except cervical with spinal curvature, malignancy, infection, or extensive fusion with complications or comorbidity.
458 - Spinal fusion except cervical with spinal curvature, malignancy, infection, or extensive fusions without comorbidities or complication or major complications or comorbidity.</td><td>450 - Single level spinal fusion except cervical with major complication or comorbidity or custom-made anatomically designed interbody fusion device.
451 - Singel level spinal fusion except cervical without major complication or comorbidity.</td><td>447 - Multiple level spinal fusion except cervical with major complication or comorbidity or custom-made anatomically designed interbody fusion device.
448 - Multiple level spinal fusion except cervical without major complication or comorbidity.</td></tr>
</table>

© 2026 Martin BI et al. *JAMA Network Open*.

eTable 2. Hierarchical Diagnosis Codes

International Classification of Diseases, version 10 (ICD-10), diagnosis codes for back and neck problems, grouped into a hierarchical classification. This grouping is an update to a previously published version based on ICD-9 codes that was validated for classifying spine surgery patients by indication. The hierarchical classification demonstrated high sensitivity and specificity. For the validation results see *Martin, B. I., et al. (2014). "Indications for spine surgery: validation of an administrative coding algorithm to classify degenerative diagnoses."* *Spine (Phila Pa 1976)* ***39****(9): 769-779.*

Indication group label	ICD-10 codes
**"Axial pain/ disc degeneration"**	Sprains/strains<br>S390, S138, S139, S134, S335, S233, S336, S135, S338, S339, S3900, S3901, S39002, S39092, S39012, S39092A, S39092S, S39092D, S138XXA, S138XXS, S138XXD, S139XXA, S139XXS, S139XXD, S134XXA, S134XXS, S134XXD, S335XXA, S335XXS, S335XXD, S233XXA, S233XXS, S233XXD, S336XXA, S336XXS, S336XXD, S135XXA, S135XXS, S135XXD, S39012A, S39012S, S39012D, S39002A, S39002S, S39002D, S338XXA, S338XXS, S338XXD, S339XXA, S339XXS, S339XXD<br>Axial pain/ disc degeneration<br>M542, M549, M539, M545, M544, M503, M548, M538, M478, M471, M472, M513, M546, M514, M479, M4642, M4643, M4646, M4647, M4649, M4641, M4648, M4640, M4644, M4645, M5450, M5442, M5441, M5440, M5033, M5031, M5032, M5030, M5489, M5136, M5137, M5134, M5135, M5459, M5382, M5383, M5386, M5387, M5381, M5388, M5380, M5384, M5385, M4789, M4712, M4713, M4716, M4711, M4710, M4714, M4715, M4722, M4723, M4726, M4727, M4721, M4728, M4720, M4724, M4725, M5146, M5147, M5144, M5145, M9903, M9904, M4781, M5451, M50321, M50322, M50323, M50320, M47892, M47893, M47896, M47897, M47891, M47898, M47899, M47894, M47895, M47812, M47813, M47816, M47817, M47811, M47818, M47819, M47814, M47815
**"Disc herniation"**	M500, M501, M509, M508, M502, M518, M512, M510, M511, S130, S330, S230, M519, M5003, M5001, M5002, M5000, M5013, M5011, M5012, M5010, M5093, M5091, M5092, M5090, M5106, M5104, M5105, M5116, M5117, M5114, M5115, M5083, M5081, M5082, M5080, M5023, M5021, M5022, M5020, M5186, M5187, M5184, M5185, M5126, M5127, M5124, M5125, M50021, M50121, M50022, M50122, M50023, M50123, M50020, M50821, M50822, M50823, M50820, M50221, M50222, M50223, M50220, M50921, M50922, M50923, M50920, S130XXA, S130XXS, S130XXD, S330XXA, S330XXS, S330XXD, S230XXA, S230XXS, S230XXD
**"Stenosis"**	M994, M993, M4802, M4803, M4806, M4807, M4801, M4808, M4800, M4804, M4805, M9973, M9963, M9953, M9933, M9923, M9943, M9956, M9961, M9974, M48062, M48061
**"Spondylolisthesis" (including vertebral dislocation and subluxation)**	S332, M434, M435, M992, M433, M431, S131, S331, S231, M991, S1320, S1329, M435X, S1245, S1235, S1215, S1265, S1255, S1225, M4312, M4313, M4316, M4317, M4319, M4311, M4318, M4310, M4314, M4315, S2311, S2316, S2317, S2312, S2313, S2314, S2315, S1310, S2310, S1244, S1234, S1214, S1264, S1254, S1224, M9912, M9913, S13111, S13121, S13141, S13151, S13161, S13171, S13181, S33111, S33121, S33131, S33141, S23111, S23161, S23163, S23171, S23121, S23123, S23133, S23141, S23143, S23151, S23153, S33101, S23101, M435X2, M435X3, M435X6, M435X7, M435X8, M435X9, M435X4, M435X5, S12450, S12350, S12150, S12650, S12550, S12250, S12451, S12351, S12151, S12651, S12551, S12251, M532X2, M532X3, M532X6, M532X7, M532X1, M532X8, M532X9, M532X4, M532X5, S13110, S13120, S13130, S13140, S13150, S13160, S13170, S13180, S33120, S33130, S33140, , S23110, S23160, S23162, S23170, S23120, S23122, S23130, S23132, S23140, S23142, S23150, S23152, S13100, S33100, S23100, S12430, S12330, S12630, S12530, S12230, S12431, S12331, S12131, S12631, S12531, S12231, S13111A, S13111S, S13111D, S13121A, S13121S, S13121D, S131131, S13131A, S13131S, S13131D, S13141A, S13141S, S13141D, S13151A, S13151S, S13151D, S13161A, S13161S, S13161D, S13171A, S13171S, S13171D, S13181A, S13181S, S13181D, S33111A, S33111S, S33111D, S33121A, S33121S, S33121D, S33131A, S33131S, S33131D, S33141A, S33141S, S33141D, S1329XA, S1329XS, S1329XD, , S332XXA, S332XXS, S332XXD, S23111A, S23111S, S23111D, S23161A, S23161S, S23161D, S23163A, S23163S, S23163D, S23171A, S23171S, S23171D, S23121A, , S23121S, S23121D, S23123A, S23123S, S23123D, S23131A, S23131S, S23131D, S23133A, S23133S, S23133D, S23141A, S23141S, S23141D, S23143A, S23143S, S23143D, S23153A, S23153S, S23153D, S13101A, S13101S, S13101D,

© 2026 Martin BI et al. *JAMA Network Open*.

	S33101A, S33101S, S33110S, S33101D, S33110D, S1320XA, S1320XS, S1320XD, S23101A, S23101S, S23101D, S23151A, S23151S, S23151D, S12450S, S12350S, S12150S, S12650S, S12550S, S12250S, S12451S, S12351S, S12151S, S12651S, , S12551S, S12251S, S13110A, S13110S, S13110D, S13120A, S13120S, S13120D, S13130A, S13130S, S13130D, S13140A, S13140S, S13140D, S13150A, S13150S, S13150D, S13160A, S13160S, S13160D, S13170A, S13170S, S13170D, S13180A, , S13180S, S13180D, S33120A, S33120S, S33120D, S33130A, S33130S, S33130D, , S33140A, S33140S, S33140D, S23110A, S23110S, S23110D, S23160A, S23160S, , S23160D, S23162A, S23162S, S23162D, S23170A, S23170S, S23170D, S23120A, S23120S, S23120D, S23122A, S23122S, S23122D, S23130A, S23130S, S23130D, S23132A, S23132S, S23132D, S23140A, S23140S, S23140D, S23142A, S23142S, S23142D, S23150A, S23150S, S23150D, S23152A, S23152S, S23152D, S13100A, , S13100D, S13100S, S33100A, S33100S, S33100D, S23100A, S23100S, S23100D, S1244XS, S1234XS, S1214XS, S1264XS, S1254XS, S1224XS, S12131S, S12430S, S12330S, S12130S, S12630S, S12530S, S12230S, S12431S, S12331S, S12631S, S12531S, S12231S
**"Scoliosis"**	M439, M403, M410, M411, M405, M414, M402, M418, M412, M401, M415, M438, M400, M404, M419, M413, , M4112, M4036, M4037, M4030, M4035, M4102, M4103, M4106, M4107, M4108, M4100, M4104, M4105, M4111, M4056, M4057, M4050, M4055, M4142, M4143, M4146, M4147, M4141, M4140, M4144, M4145, M4182, M4183, M4186, M4187, M4180, M4184, M4185, M4122, M4123, M4126, M4127, M4120, M4124, M4125, M4029, M4012, M4013, M4010, M4014, M4015, M4152, M4153, M4156, M4157, M4150, M4154, M4155, M4003, M4000, M4004, M4005, M438X, M4046, M4047, M4040, M4045, M4130, M4134, M4135, M4020, M4157, M41122, M41123, M41126, M41127, M41129, M41124, M41125, M41112, M41113, M41116, M41117, M41119, M41114, M41115, M40292, M40293, M40299, M40294, M40295, M438X2, M438X3, M438X6, M438X7, M438X1, M438X8, M438X9, M438X4, M438X5, M40202, M40203, M40209, M40204, M40205
**"Sciatica Radiculopathy myelopathy spinal nerve problem or brachial plexus problem"**	M544, M471, M472, M543, M541, M500, M501, M510, M511, M994, M993, M433, M992, M531, M530, G542, G544, G543, S142, G540, S143, S145, S344, S242, S148, S144, S149, G541, M5442, M5441, M5440, M4712, M4713, M4716, M4711, M4710, M4714, M4715, M4722, M4723, M4726, M4727, M4721, M4728, , M4720, M4724, M4725, M5003, M5001, M5002, M5013, M5011, M5012, M5010, M5106, M5104, M5105, M5116, M5117, M5114, M5115, M5412, M5413, M5416, M5417, M5411, M5418, M5410, , M5414, M5415, M5432, M5431, M5430, M5000, S3421, S3422, S1980, M21371, M21372, M50021, M50121, M50022, M50122, M50023, M50123, M50020, M48062, S142XXA, S142XXD, S143XXA, S143XXS, S143XXD, S145XXA, S145XXS, S145XXD, S344XXA, S344XXS, S344XXD, S142XXS, S3421XA, S3421XS, S3421XD, S3422XA, S3422XS, S3422XD, S242XXA, S242XXS, S242XXD, S148XXA, S148XXS, S148XXD, S144XXA, S144XXS, S144XXD, S149XXA, S149XXS, S149XXD, S1980XA, S1980XS, S1980XD

© 2026 Martin BI et al. *JAMA Network Open*.

**“Other”**<br><br>**(Non-degenerative diagnoses)**	**Fracture**<br><br>M485, M484, S322, S124, S120, S123, S129, S128, S121, S126, S122, M430, M4852, M4853, M4856, M4857, M4851, M4858, M4850, M4854, M4855, M4842, M4843, M4846, M4847, M4841, M4848, M4840, M4844, M4845, S2200, S1204, S1212, S1249, S1209, S1239, S3219, S1219, S1269, S1259, S1229, S1203, M4302, M4303, M4306, M4307, M4309, M4301, M4308, M4300, M4304, M4305, S1201, S3214, S3215, S3216, S3217, S1211, S1240, S1230, S3210, S1210, S1260, S1250, S1220, S1202, S12110, S12040, S12030, S32111, S32121, S32131, S12041, S12031, S12112, S32110, S32120, S32130, S12120, S12490, S12090, S12390, S12190, S12690, S12590, S12290, S32058, S32018, S22018, S22048, S32048, S32028, S22028, S22088, S22068, S22078, S32038, S22038, S32008, S22008, S12121, S12491, S12091, S12391, S12191, S12691, S12591, S12291, S12111, S32112, S32122, S32132, S32051, S32011, S22011, S32041, S22041, S32121, S22021, S22081, S22051, S22061, S22071, S32031, S22031, S32001, S22001, S22002, S12400, S12000, S12300, S12100, S12600, S12500, S12200, S32059, S32019, S22019, S32049, S22049, S32029, S22089, S22059, S22069, S22029, S22079, S32039, S22039, S32009, S22009, S12401, S12001, S12301, S12101, S12601, S12501, S12201, S12130, S32119, S32129, S32139, S32052, S32012, S22012, S32042, S22042, S32022, S22022, S22082, S22052, S22058, S22062, S22072, S32032, S22032, S32002, S32050, S32010, S22010, S32040, S22040, S32020, S22020, S22080, S22050, S22060, S22070, S32030, S22030, S32000, S22000, S12110A, S12110B, S12110S, S12110G, S12110K, S12110D, M4852XA, M4852XS, M4852XG, M4852XD, M4853XA, M4853XS, M4853XG, M4853XD, M4854XD, M4856XA, M4856XS, M4856XG, M4856XD, M4857XA, M4857XS, M4857XG, M4857XD, M4851XA, M4851XS, M4851XG, M4851XD, M4858XA, M4858XS, M4858XG, M4858XD, M4850XA, M4850XD, M4850XS, M4850XG, M4854XA, M4854XS, M4854XG, M4855XA, M4855XS, M4855XG, M4855XD, S12040A, S12040B, S12040S, S12040G, S12040K, S12040D, S12030A, S12030B, S12030S, S12030G, S12030K, S12030D, M4842XA, M4842XS, M4842XG, M4842XD, M4843XA, M4843XS, M4843XG, M4843XD, M4846XA, M4846XS, M4846XG, M4846XD, M4847XA, M4847XS, M4847XG, M4847XD, M4841XA, M4841XS, M4841XG, M4841XD, M4848XA, M4848XS, M4848XG, M4848XD, M4840XA, M4840XS, M4840XG, M4840XD, M4844XA, M4844XS, M4844XG, M4844XD, M4845XA, M4845XS, M4845XG, M4845XD, S322XXA, S322XXB, S322XXS, S322XXG, S322XXK, S322XXD, S129XXA, S129XXS, S129XXD, S128XXA, S128XXS, S128XXD, S32111A, S32111B, S32111S, S32111G, S32111K, S32111D, S32121A, S32121B, S32121S, S32121G, S32121K, S32121D, S32131A, S32131B, S32131S, S32131G, S32131K, S32131D, S12041A, S12041B, S12041S, S12041G, S12041K, S12041D, S12031A, S12031B, S12031S, S12031G, S12031K, S12031D, S12112A, S12112B, S12112S, S12112G, S12112K, S12112D, S32110A, S32110B, S32110S, S32110G, S32110K, S32110D, S32120A, S32120B, S32120S, S32120G, S32120K, S32120D, S32130A, S32130B, S32130S, S32130G, S32130K, S32130D, S12120A, S12120B, S12120S, S12120G, S12120K, S12120D, S12490A, S12490B, S12490S, S12490G, S12490K, S12490D, S12090A, S12090B, S12090S, S12090G, S12090K, S12090D, S12390A, S12390B, S12390S, S12390G, S12390K, S12390D, S12190A, S12190B, S12190S, S12190G,

© 2026 Martin BI et al. *JAMA Network Open*.

	S12190K, S12190D, S12690A, S12690B, S12690S, S12690G, S12690K, S12690D, S12590A, S12590B, S12590S, S12590G, S12590K, S12590D, S12290A, S12290B, S12290S, S12290G, S12290K, S12290D, S32058A, S32058B, S32058S, S32058G, S32058K, S32058D, S32018A, S32018B, S32018S, S32018G, S32018K, S32018D, S22018A, S22018B, S22018S, S22018G, S22018K, S22018D, S32048A, S32048B, S32048S, S32048G, S32048K, S32048D, S22048A, S22048B, S22048S, S22048G, S22048K, S22048D, S3219XA, S3219XB, S3219XS, S3219XG, S3219XK, S3219XD, S32028A, S32028B, S32028S, S32028G, S32028K, S32028D, S22028A, S22028B, S22028S, S22028G, S22028K, S22028D, S22088A, S22088B, S22088S, S22088G, S22088K, S22088D, S22058B, S22058S, S22058G, S22058K, S22058D, S22068A, S22068B, S22068S, S22068G, S22068K, S22068D, S22078A, S22078B, S22078S, S22078G, S22078K, S22078D, S32038A, S32038B, S32038S, S32038G, S32038K, S32038D, S22038A, S22038B, S22038S, S22038G, S22038K, S22038D, S32008A, S32008B, S32008S, S32008G, S32008K, S32008D, S22008A, S22008B, S22008S, S22008G, S22008K, S22008D, S12121A, S12121B, S12121S, S12121G, S12121K, S12121D, S12491A, S12491B, S12491S, S12491G, S12491K, S12491D, S12091A, S12091B, S12091S, S12091G, S12091K, S12091D, S12391A, S12391B, S12391S, S12391G, S12391K, S12391D, S12191A, S12191B, S12191S, S12191G, S12191K, S12191D, S12691A, S12691B, S12691S, S12691G, S12691K, S12691D, S12591A, S12591B, S12591S, S12591G, S12591K, S12591D, S12291A, S12291B, S12291S, S12291G, S12291K, S12291D, S12450A, S12450B, S12450G, S12450K, S12450D, S12350A, S12350B, S12350G, S12350K, S12350D, S12150A, S12150B, S12150G, S12150K, S12150D, S12650A, S12650B, S12650G, S12650K, S12650D, S12550A, S12550B, S12550G, S12550K, S12550D, S12250A, S12250B, S12250G, S12250K, S12250D, S12451A, S12451B, S12451G, S12451K, S12451D, S12351A, S12351B, S12351G, S12351K, S12351D, S12151A, S12151B, S12151G, S12151K, S12151D, S12651A, S12651B, S12651G, S12651K, S12651D, S12551A, S12551B, S12551G, S12551K, S12551D, S12251A, S12251B, S12251G, S12251K, S12251D, S12111A, S12111B, S12111S, S12111G, S12111K, S12111D, S32112A, S32112B, S32112S, S32112G, S32112K, S32112D, S32122A, S32122B, S32122S, S32122G, S32122K, S32122D, S32132A, S32132B, S32132S, S32132G, S32132K, S32132D, S32051A, S32051B, S32051S, S32051G, S32051K, S32051D, S1201XA, S1201XB, S1201XS, S1201XG, S1201XK, S1201XD, S32051A, S32051B, S32051S, S32051G, S32051K, S32051D, S1201XA, S1201XB, S1201XS, S1201XG, S1201XK, S1201XD, S32011A, S32011B, S32011S, S32011G, S32011K, S32011D, S22011A, S22011B, S22011S, S22011G, S22011K, S22011D, S32041A, S32041B, S32041S, S32041G, S32041K, S32041D, S22041A, S22041B, S22041S, S22041G, S22041K, S22041D, S32021A, S32021B, S32021S, S32021G, S32021K, S32021D, S22021A, S22021B, S22021S, S22021G, S22021K, S22021D, S22081A, S22081B, S22081S, S22081G, S22081K, S22081D, S22051A, S22051B, S22051S, S22051G, S22051K, S22051D, S22061A, S22061B, S22061S, S22061G, S22061K, S22061D, S22071A, S22071B, S22071S, S22071G, S22071K, S22071D, S32031A, S32031B, S32031S, S32031G, S32031K, S32031D, S22031A, S22031B, S22031S, S22031G, S22031K, S22031D, S32001A, S32001B, S32001S, S32001G, S32001K, S32001D, S22001A, S22001B, S22001S, S22001G, S22001K, S22001D, S3214XA, S3214XB, S3214XS, S3214XG, S3214XK, S3214XD, S3215XA, S3215XB, S3215XS, S3215XG, S3215XK, S3215XD, S3216XA, S3216XB, S3216XS, S3216XG, S3216XK, S3216XD, S3217XA, S3217XB, S3217XS, S3217XG, S3217XK, S3217XD, S1244XA, S1244XB, S1244XG, S1244XK, S1244XD, S1234XA, S1234XB, S1234XG, S1234XK, S1234XD, S1214XA, S1214XB, S1214XG, S1214XK, S1214XD, S1264XA, S1264XB, S1264XG, S1264XK, S1264XD, S1254XA, S1254XB, S1254XG, S1254XK, S1254XD, S1224XA, S1224XB, S1224XG, S1224XK, S1224XD, S12400A, S12400B, S12400S, S12400G, S12400K, S12400D, S12000A, S12000S, S12000G, S12000K, S12000D, S12300A, S12300B, S12300S, S12300G, S12300K, S12300D, S12100A, S12100B, S12100S, S12100G, S12100K, S12100D, S12600A, S12600B, S12600S, S12600G, S12600K, S12600D, S12500A, S12500B, S12500S, S12500G, S12500K, S12500D, S12200A, S12200B, S12200S, S12200G, S12200K, S12200D, S32059A, S32059B, S32059S, S32059G, S32059K, S32059D, S32019A, S32019B, S32019S, S32019G, S32019K, S32019D, S22019B, S22019A, S22019S, S22019G, S22019K, S22019D, S32049A, S32049B, S32049S, S32049G, S32049K, S32049D, S22049A, S22049B, S22049S, S22049G, S22049K, S22049D, S3210XA, S3210XB, S3210XS, S3210XG, S3210XK, S3210XD, S32029A, S32029B, S32029S, S32029G, S32029K, S32029D, S22029A, S22029B, S22029S, S22029G, S22029K, S22029D, S22089A, S22089B, S22089S, S22089G, S22089K, S22089D, S22059A, S22059B, S22059S, S22059G, S22059K, S22059D, S22069A, S22069B, S22069S, S22069G, S22069K, S22069D, S22079A, S22079B, S22079S, S22079G, S12000B, S22079K, S22079D, S32039A, S32039B, S32039S, S32039G, S32039K, S32039D, S22039A, S22039B, S22039S, S22039G, S22039K, S22039D, S32009A, S32009B, S32009S, S32009G, S32009K, S32009D, S22009A, S22009B, S22009S, S22009G, S22009K, S22009D, S12401A, S12401B, S12401S, S12401G, S12401K, S12401D, S12001A, S12001B, S12001S, S12001G, S12001K, S12001D, S12301A, S12301B, S12301S, S12301G, S12301K, S12301D, S12101A, S12101B, S12101S, S12101G, S12101K, S12101D, S12601A, S12601B, S12601S, S12601G, S12601K, S12601D, S12501A, S12501B, S12501S, S12501G, S12501K, S12501D, S12201A, S12201B, S12201S, S12201G, S12201K, S12201D, S12430A, S12430B, S12430G, S12430K, S12430D, S12330A, S12330B, S12330G, S12330K, S12330D, S12130A, S12130B, S12130G, S12130K, S12130D, S12630A, S12630B, S12630G, S12630K, S12630D, S12530A, S12530B, S12530G, S12530K, S12530D, S12230A, S12230B, S12230G, S12230K, S12230D, S12431A, S12431B, S12431G, S12431K, S12431D, S12331A, S12331B, S12331G,

© 2026 Martin BI et al. *JAMA Network Open*.

	S12331K, S12331D, S12131A, S12131B, S12131G, S12131K, S12131D, S12631A, S12631B, S12631G, S12631K, S12631D, S12531A, S12531B, S12531G, S12531K, S12531D, S12231A, S12231B, S12231G, S12231K, S12231D, S32119A, S32119B, S32119S, S32119G, S32119K, S32119D, S32129A, S32129B, S32129S, S32129G, S32129K, S32129D, S32139A, S32139B, S32139S, S32139G, S32139K, S32139D, S32052A, S32052B, S32052S, S32052G, S32052K, S32052D, S1202XA, S1202XB, S1202XS, S1202XG, S1202XK, S1202XD, S32012A, S32012B, S32012S, S32012G, S32012K, S32012D, S22012A, S22012B, S22012S, S22012G, S22012K, S22012D, S32042A, S32042B, S32042S, S32042G, S32042K, S32042D, S22042A, S22042B, S22042S, S22042G, S22042K, S22042D, S32022A, S32022B, S32022S, S32022G, S32022K, S32022D, S22022A, S22022B, S22022S, S22022G, S22022K, S22022D, S22082A, S22082B, S22082S, S22082G, S22082K, S22082D, S22052A, S22052B, S22052S, S22058A, S22052G, S22052K, S22052D, S22062A, S22062B, S22062S, S22062G, S22062K, S22062D, S22072A, S22072B, S22072S, S22072G, S22072K, S22072D, S32032A, S32032B, S32032S, S32032G, S32032K, S32032D, S22032A, S22032B, S22032S, S22032G, S22032K, S22032D, S32002A, S32002B, S32002S, S32002G, S32002K, S32002D, S22002A, S22002B, S22002S, S22002G, S22002K, S22002D, S32050A, S32050B, S32050S, S32050G, S32050K, S32050D, S32010A, S32010B, S32010S, S32010G, S32010K, S32010D, S22010A, S22010B, S22010S, S22010G, S22010K, S22010D, S32040A, S32040B, S32040S, S32040G, S32040K, S32040D, S22040A, S22040B, S22040S, S22040G, S22040K, S22040D, S32020A, S32020B, S32020S, S32020G, S32020K, S32020D, S22020A, S22020B, S22020S, S22020G, S22020K, S22020D, S22080A, S22080B, S22080S, S22080G, S22080K, S22080D, S22050A, S22050B, S22050S, S22050G, S22050K, S22050D, S22060A, S22060B, S22060S, S22060G, S22060K, S22060D, S22070A, S22070B, S22070S, S22070G, S22070K, S22070D, S32030A, S32030B, S32030S, S32030G, S32030K, S32030D, S22030A, S22030B, S22030S, S22030G, S22030K, S22030D, S32000A, S32000B, S32000S, S32000G, S32000K, S32000D, S22000A, S22000B, S22000S, S22000G, S22000K, S22000D<br><br>**Spinal Cord Injury**<br><br>M531, M530, M470, S140, S340, S240, S2411, S3401, S3402, S1410, S2410, S14131, S14132, S14133, S14134, S14135, S14136, S14137, S14138, S24131, S24134, S24132, S24133, S14139, S24139, M47012, M47013, M47016, M47011, M47019, M47014, M47015, S14141, S14142, S14143, S14144, S14145, S14146, S14147, S14148, S24141, S24144, S24142, S24143, S14149, S24149, S14121, S14122, S14123, S14124, S14125, S14126, S14127, S14128, S14129, S14111, S14112, S14113, S14114, S14115, S14116, S14117, S14118, S24111, S14119, S34111, S34112, S34113, S34114, S34115, S34131, S34119, S34121, S34122, S34123, S34124, S34125, S34132, S34129, S14151, S14152, S14153, S14154, S14155, S14156, S14157, S14158, S24151, S24154, S24152, S24153, S14159, S24159, S14101, S14102, S34101, S34102, S34103, S34104, S34105, S34139, S34109, M47022, M47021, M47029, S14103, S14104, S14105, S14106, S14107, S14108, S24101, S14109, S14131A, S14131S, S14131D, S14132A, S14131S, S14131D, S14133A, S14133S, S14133D, S14134A, S14134S, S14134D, S14135A, S14135S, S14135D, S14136A, S14136S, S14136D, S14137A, S14137S, S14137D, S14138A, S14138S, S14138D, S24131A, S24131S, S24131D, S24134A, S24134S, S24134D, S24132A, S24132D, S24133A, S24132S, S24133D, S24133S, S14139A, S14139S, S14139D, S24139A, S24139S, S24139D, S14141A, S14141S, S14141D, S14142A, S14142S, S14142D, S14143A, S14143S, S14143D, S14144A, S14144S, S14144D, S14145A, S14145S, S14145D, S14146A, S14146S, S14146D, S14147A, S14147S, S14147D, S14148A, S14148S, S14148D, S24141A, S24141S, S24141D, S24144A, S24144S, S24144D, S24142A, S24142S, S24142D, S24143A, S24143S, S24143D, S14149A, S14149S, S14149D, S24149A, S24149S, S24149D, S14121A, S14121S, S14121D, S14122A, S14122S, S14122D, S14123A, S14123S, S14123D, S14124A, S14124S, S14124D, S14125A, S14125S, S14125D, S14126A, S14126S, S14126D, S14127A, S14127S, S14127D, S14128A, S14128S, S14128D, S14129A, S14129S, S14129D, S14111A, S14111S, S14111D, S14112A, S14112S, S14112D, S14113A, S14113S, S14113D, S14114A, S14114S, S14114D, S14115A, S14115S, S14115D, S14116A, S14116S, S14116D, S14117A, S14117S, S14117D, S14118A, S14118S, S14118D, S24111A, S24111S, S24111D, S24114A, S24114S, S24114D, S24112A, S24112S, S24112D, S24113A, S24113S, S24113D, S14119A, S14119S, S14119D, S24119A, S24119S, S24119D, S34111A, S34111S, S34111D, S34112A, S34112S, S34112D, S34113A, S34113S, S34113D, S34114A, S34114S, S34114D, S34115A, S34115S, S34115D, S34131A, S34131S, S34131D, S34119A, S34119S, S34119D, S140XXA, S140XXS, S140XXD, S3401XA, S3401XS, S3401XD, S3402XA, S3402XS, S3402XD, S240XXA, S240XXS, S240XXD, S34121A, S34121S, S34121D, S34122A, S34122S, S34122D, S34123A, S34123S, S34123D, S34124A, S34124S, S34124D, S34125A, S34125S, S34125D, S34132A, S34132S, S34132D, S34129A, S34129S, S34129D, S14151A, S14151S, S14151D, S14152A, S14152S, S14152D, S14153A, S14153S, S14153D, S14154A, S14154S, S14154D, S14155A, S14155S, S14155D, S14156A, S14156S, S14156D, S14157A, S14157S, S14157D, S14158A, S14158S, S14158D, S24151A, S24151S, S24151D, S24154A, S24154S, S24154D, S24152A, S24152S, S24152D, S24153A, S24153S, S24153D, S14159A, S14159S, S14159D, S24159A, S24159S, S24159D, S14101A, S14101S, S14101D, S14102A, S14102S, S14102D, S14103A, S14103S, S14103D, S14104A, S14104S, S14104D, S14105A, S14105S, S14105D, S14106A, S14106S, S14106D, S14107A, S14107S, S14107D, S14108A, S14108S, S14108D, S24101A, S24101S, S24101D, S24104A, S24104S, S24104D, S24102A,

© 2026 Martin BI et al. *JAMA Network Open*.

	S24102S, S24102D, S24103A, S24103S, S24103D, S14109A, S14109S, S14109D, S24109A, S24109S, S24109D, S34101A, S34101S, S34101D, S34102A, S34102S, S34102D, S34103A, S34103S, S34103D, S34104A, S34104S, S34104D, S34105A, S34105S, S34105D, S34139A, S34139S, S34139D, S34109A, S34109S, S34109D
	**Congenital or other anomaly**<br><br>Q76, G542, G544, G543, Q675, Q763, Q762, S343, G061, Q761, Q764, Q058, Q760, M429, M436, M881, M4212, M4213, M4216, M4217, M4219, M4211, M4218, M4210, M4214, M4215, M4812, M4813, M4816, M4817, M4819, M4811, M4818, M4810, M4814, M4815, Q7641, Q7642, M4322, M4323, M4326, M4327, M4321, M4328, M4320, M4324, M4325, M4202, M4203, M4206, M4207, M4209, M4201, M4208, M4200, M4204, M4205, M4822, M4823, M4826, M4827, M4821, M4820, M4824, M4825, S1182, M9983, M9984, Q7649, M4832, M4833, M4836, M4837, M4831, M4838, M4830, M4834, M4835, M2578, M4327, M1468, Q76412, Q76413, Q76411, Q76414, Q76415, Q76419, Q76426, Q76427, Q76428, Q76425, Q76429, S343XXA, S343XXS, S343XXD, S1182XA, S1182XS, S1182XD
	**Inflammatory spondylopathy discitis**<br><br>M456, M452, M453, M457, M450, M451, M454, M455, M459, M458, M462, M461, M489, M4622, M4623, M4626, M4627, M4621, M4628, M4620, M4624, M4625, M4682, M4683, M4686, M4687, M4689, M4681, M4688, M4680, M4684, M4685, M5402, M5403, M5406, M5407, M5401, M5408, M5400, M5404, M5405, M5409, M4602, M4603, M4606, M4607, M4609, M4601, M4608, M4600, M4604, M4605, M4982, M4983, M4986, M4987, M4989, M4981, M4988, M4980, M4984, M4985, M4692, M4693, M4696, M4697, M4699, M4691, M4698, M4690, M4694, M4695, M488X2, M488X3, M488X6, M488X7, M488X1, M488X8, M488X9, M488X4, M488X5,
	**Pathological fracture due to osteoporosis or neoplasm**<br><br>M810, M816, M808, M818, M8000, M8008, M8080, M8458, M8450, M8448, M8440, M8000XA, M8000XS, M8000XG, M8000XP, M8000XK, M8000XD, M8008XA, M8008XS, M8008XG, M8008XP, M8008XK, M8008XD, M8080XA, M8080XS, M8080XG, M8080XP, M8080XK, M8080XD, M8088XA, M8088XS, M8088XG, M8088XP, M8088XK, M8088XD, M8458XA, M8458XS, M8458XG, M8458XP, M8458XK, M8458XD, M8450XA, M8450XS, M8450XG, M8450XP, M8450XK, M8450XD, M8448XA, M8448XS, M8448XG, M8448XP, M8448XK, M8448XD, M8440XA, M8440XS, M8440XG, M8440XP, M8440XK, M8440XD
	**Surgical aftercare**<br><br>Z981, M961, M960, T8463, T85192, T8463XA, T8463XS, T8463XD, T85192A, T85192S, T85192D, T84226A, T84216A, T84296A, T8485XA
	**Infection of intervertebral disc; infective spondylopathy**<br><br>M463, M4632, M4633, M4636, M4637, M4639, M4631, M4638, M4630, M4634, M4635, M4652, M4653, M4656, M4657, M4659, M4651, M4658, M4650, M4654, M4655

© 2026 Martin BI et al. *JAMA Network Open*.

eTable 3. Annual Volume of Hospital-Owned Outpatient Departments Lumbar Surgery (Fusion and Nonfusion) Among Patients Aged 20 Years or Older, by *Current Procedural Terminology* Codes
Based on analysis of Nationwide Ambulatory Surgical Sample, 2016-2022.

YEAR	CPT 22612: Arthrodesis, posterior or posterolateral technique, single interspace; lumbar (with lateral transverse technique, when performed)	CPT 22630: Arthrodesis, posterior interbody technique, including laminectomy and/or discectomy to prepare interspace (other than for decompression), single interspace; lumbar	CPT 22633: Arthrodesis, combined posterior or posterolateral technique with posterior interbody technique, including laminectomy and/or discectomy sufficient to prepare interspace (other than for decompression); single interspace and segment, lumbar (do not report with 22612 or 22630 at the same level)	Total lumbar fusion episodes as defined by TEAM	Fusions based on other CPT codes not included by TEAM	Total lumbar fusions †	Lumbar decompression without fusion ‡
2016	2936	1064	1146	5191	941	6132	202536
2017	5250	1344	903	7540	999	8539	199650
2018	6345	1284	582	8307	1148	9454	194817
2019	7074	1113	497	8813	1242	10054	205833
2020	8968	1464	2435	12964	1543	14507	182666
2021	17809	5794	5678	29357	6745	36102	189652
2022	15210	5952	2316	23525	3806	27331	192263

Source: Nationwide Ambulatory Surgery Sample, 2016-2022; CPT "American Medical Association's Current Procedural Terminology Codes"; TEAM "Transforming Episode Accountability Model" Medicare bundled payment model, only includes fusions with CPT codes 22612, 22630, 22633

† Additional non-TEAM fusion-related CPT codes include 22845, 22808, 22800, 22810, 22842, 22843, 22840, 22841, 22849, 22850, 22846, 22854, 22812, 22847, 22804, 22534, 22854, 22854, 22548, 22854, 22854, 22612, 22630, 22854, 22586, 22854, 0775T, 22854, 22854, 22556, 22610, 22614, 22633, 22854, 22854.

‡ Lumbar decompression without fusion are identified by CPT codes for laminectomy, laminotomy or discectomy (CPT 63015, 63017, 63016, 63035, 63043, 63076, 63082, 63044, 63053, 63091, 63078, 63086, 63088, 63048, 22100, 22101, 22102, 63001, 63020, 63040, 63045, 63050, 63051, 63075, 63081, 63005, 63012, 63030, 63042, 63047, 63052, 63056, 63090, 63011, 63003, 63046, 63055, 63064, 63066, 63077, 63085, 63057, 63087, 62380, 63172, 63173), distraction (CPT 22870, 22868, 22869, 22867), or Excision (CPT 63295, 63308, 21899, 63250, 63265, 63270, 63275, 63280, 63285, 63300, 63304, 21920, 21925, 21930, 21931, 63267, 63272, 63277, 63282, 63303, 63307, 63268, 63273, 63278, 63283, 63251, 63266, 63271, 63276, 63281, 63286, 63301, 63305, 63302, 63306, 63252, 63287, 63290) without coded fusion procedures.

© 2026 Martin BI et al. *JAMA Network Open*.

eTable 4. Annual Inflation Adjusted Mean Cost Per Case and Total Hospital Costs (ie, "National Bill") for Inpatient Lumbar Fusion, 2002-2023

All costs are adjusted for inflation using the GDP implicit price deflator, reported as 2023 USD equivalents, and adjusting for age, sex, race, surgical indication, Charlson comorbidity index, osteoarthritis, osteoporosis.

Annual mean cost per case and total aggregate ("national bill" in Billions) for hospital cost of inpatient lumbar fusion				
**YEAR**	**Mean**	**95% CI**	**Total ($Billion)**	**95% CI**
2002	$25,849	($25,684 - $26,015)	$3.86	($3.81 - $3.92)
2003	$29,376	($29,175 - $29,577)	$4.54	($4.48 - $4.61)
2004	$30,595	($30,414 - $30,776)	$4.88	($4.81 - $4.95)
2005	$33,885	($33,690 - $34,081)	$6.05	($5.96 - $6.14)
2006	$34,065	($33,878 - $34,253)	$5.83	($5.75 - $5.92)
2007	$36,461	($36,263 - $36,658)	$6.51	($6.41 - $6.60)
2008	$37,797	($37,595 - $38,000)	$7.99	($7.89 - $8.10)
2009	$37,933	($37,731 - $38,135)	$8.43	($8.31 - $8.54)
2010	$41,086	($40,873 - $41,298)	$9.97	($9.84 - $10.1)
2011	$39,742	($39,533 - $39,951)	$9.73	($9.60 - $9.85)
2012	$38,129	($37,919 - $38,340)	$8.94	($8.82 - $9.06)
2013	$37,972	($37,763 - $38,180)	$9.14	($9.02 - $9.26)
2014	$37,389	($37,195 - $37,583)	$9.29	($9.17 - $9.42)
2015	$40,449	($40,234 - $40,664)	$10.6	($10.5 - $10.8)
2016	$48,649	($48,378 - $48,919)	$14.9	($14.7 - $15.1)
2017	$48,443	($48,183 - $48,702)	$14.9	($14.7 - $15.1)
2018	$47,399	($47,152 - $47,647)	$14.8	($14.6 - $15.0)
2019	$49,127	($48,854 - $49,399)	$15.7	($15.5 - $15.9)
2020	$51,362	($51,059 - $51,665)	$14.5	($14.3 - $14.7)
2021	$49,572	($49,281 - $49,864)	$13.9	($13.7 - $14.1)
2022	$47,136	($46,855 - $47,418)	$13.7	($13.5 - $13.9)
2023	$45,458	($45,207 - $45,709)	$14.1	($13.9 - $14.2)
% change 2002-2023	75.9%		265.3%	

© 2026 Martin BI et al. *JAMA Network Open*.

eTable 5. Annual Trends in Mean Inpatient Hospital Cost for Lumbar Fusion and Nonfusion Operations, by Diagnosis Related Groups (2002-2015) and 2025 Revised Diagnosis Related Groups That Separate 1-Level and Multilevel Procedures (2016-2023)
All costs are adjusted for inflation using the GDP implicit price deflator, reported as 2023 USD equivalents, and adjusting for age, sex, race, surgical indication, Charlson comorbidity index, osteoarthritis, osteoporosis.

Year	Non-fusion Cost (95% CI)	Anterior-Posterior (AP) Cost (95%CI)		Complex Cost (95%CI)	Single column Cost (95%CI)	
2002	$8,464 ($8,408 – $8,521)	$40,743 ($39,672 - $41,813)			$24,515 ($24,361 - $24,669)	
2003	$9,245 ($9,185 – $9,306)	$41,130 ($40,202 - $42,057)			$27,994 ($27,796 -$28,193)	
2004	$9,495 ($9,435 – $9,555)	$41,013 ($40,135 - $41,891)			$29,063 ($28,891 - $29,235)	
2005	$9,998 ($9,933 – $10,063)	$56,701 ($54,989 - $58,414)		$48,860 ($44,668 - $53,052)	$32,280 ($32,099 - $32,462)	
2006	$10,232 ($10,165 – $10,299)	$51,261 ($49,989 - $52,533)		$45,824 ($43,627 - $48,021)	$32,413 ($32,234 -$32,592)	
2007	$10,904 ($10,826 – $10,981)	$54,812 ($53,460 - $56,164)		$50,534 ($48,139 - $52,929)	$34,636 ($34,450 - $34,821)	
2008	$11,673 ($11,590 – $11,755)	$57,646 ($56,234 - $59,057)		$63,333 ($60,762 - $65,905)	$35,687 ($34,497 - $35,877)	
2009	$11,392 ($11,302 – $11,481)	$60,329 ($58,900 - $61,757)		$65,081 ($62,633 - $67,528)	$35,510 ($35,325 - $35,694)	
2010	$12,848 ($12,750 – $12,946)	$61,375 ($60,103 - $62,647)		$68,823 ($66,626 - $71,021)	$38,382 ($38,185 - $38,580)	
2011	$13,505 ($13,405 – $13,605)	$61,565 ($60,421 -$62,709)		$68,492 ($66,042 - $70,944)	$36,857 ($36,663 - $37,051)	
2012	$13,442 ($13,330 – $13,555)	$62,381 ($60,943 -$63,820)		$61,346 ($59,100 - $63,591)	$35,433 ($35,245 - $35,622)	
2013	$13,487 ($13,377 – $13,597)	$62,379 ($60,946 - $63,813)		$62,786 ($60,658 - $64,914)	$35,123 ($34,939 - $35,307)	
2014	$14,059 ($13,937 – $14,181)	$61,104 ($59,879 - $62,334)		$64,454 ($62,336 - $66,572)	$34,494 ($34,322 - $34,666)	
2015	$15,047 ($14,911 – $15,182)	$61,961 ($60,631 -$ 63,291)		$65,369 ($63,020 - $67,718)	$34,452 ($34,254 - $34,650)	
	**Non-fusion Cost (95%CI)**	**AP 1-level Cost (95%CI)**	**AP 2-7 level Cost (95%CI)**	**Complex Cost (95%CI)**	**1-column 1-level Cost (95%CI)**	**1-column 2-7 level Cost (95%CI)**
2016	$16,144 ($15,964 - $16,324)	$45,137 ($44,477 - $45,798)	$66,610 ($65,444 - $67,776)	$76,341 ($75,114 – $77,568)	$37,660 ($37,387 - $37,933)	$53,301 ($52,755 - $53,747)

© 2026 Martin BI et al. *JAMA Network Open*.

**2017**	$17,005 ($16,808 - $17,202)	$43,512 ($42,994 - $44,031)	$65,399 ($64,416 - $66,381)	$75,104 ($74,006 – $76,201)	$37,041 ($36,767 - $37,314)	$52,707 ($52,167 - $53,246)
**2018**	$17,514 ($17,287 - $17,740)	$40,734 ($40,361 - $41,107)	$62,253 ($61,479 - $63,027)	$71,465 ($70,498 – $72,432)	$35,444 ($35,155 - $35,734)	$50,346 ($49,790 - $50,902)
**2019**	$18,678 ($18,445 - $18,911)	$41,143 ($40,774 - $41,512)	$62,246 ($61,497 - $62,995)	$73,387 ($72,373 – $74,401)	$36,416 ($36,075 - $36,756)	$52,737 ($52,080 - $53,395)
**2020**	$20,305 ($20,029 - $20,580)	$42,362 ($41,967 - $42,756)	$63,724 ($62,941 - $64,506)	$76,038 ($74,949 – $77,126)	$37,576 ($37,190 - $37,962)	$54,828 ($54,066 - $55,589)
**2021**	$20,353 ($20,094 - $20,613)	$40,231 ($39,855 - $40,608)	$61,461 ($60,751 - $62,171)	$72,056 ($71,063 – $73,049)	$36,611 ($36,210 - $37,013)	$52,562 ($51,843 - $53,281)
**2022**	$20,733 ($20,458 - $21,009)	$37,897 ($37,557 - $38,237)	$58,371 ($57,695 - $59,048)	$68,029 ($67,147 – $68,911)	$34,421 ($34,002 - $34,840)	$50,549 ($49,819 - $51,278)
**2023**	$21,225 ($20,955 - $21,495)	$36,071 ($35,743 - $36,400)	$55,034 ($54,420 – $55,650)	$65,308 ($64,518 - $66,098)	$33,610 ($33,178 - $34,042)	$48,931 ($48,315 - $49,547)

© 2026 Martin BI et al. *JAMA Network Open*.

eTable 6. Annual Trends in Rates (per 100 000) of Inpatient Discharges for Lumbar Fusion, by Diagnosis Related Groups (2002-2015) and 2025 Revised Diagnosis Related Groups That Separate 1-Level and Multilevel Procedures (2016-2023)

	Anterior-Posterior (AP)		Complex		Single column	
**Year**	**Volume**	**Rate per 100,000 (95%CI)**	**Volume**	**Rate per 100,000 (95%CI)**	**Volume**	**Rate per 100,000 (95%CI)**
**2002**	7,991	2.96 (2.89–3.02)			140,832	57.28 (56.97–57.59)
**2003**	10,803	3.92 (3.84–4.00)			143,852	57.14 (56.84–57.45)
**2004**	13,579	4.87 (4.79–4.96)			144,150	55.96 (55.66–56.26)
**2005**	6,951	2.42 (2.36–2.47)	652	0.24 (0.22–0.25)	169,521	64.52 (64.20–64.85)
**2006**	8,252	2.82 (2.76–2.88)	2,733	0.97 (0.93–1.01)	163,208	63.27 (62.95–63.58)
**2007**	8,794	2.97 (2.90–3.03)	3,429	1.21 (1.17–1.25)	168,312	64.39 (64.07–64.71)
**2008**	9,776	3.33 (3.27–3.40)	5,134	1.84 (1.79–1.89)	199,232	76.77 (76.43–77.12)
**2009**	11,465	3.71 (3.64–3.78)	5,481	1.85 (1.80–1.90)	207,673	76.39 (76.05–76.73)
**2010**	15,831	5.00 (4.92–5.08)	6,524	2.17 (2.11–2.22)	222,006	80.04 (79.69–80.38)
**2011**	15,959	4.97 (4.89–5.05)	7,713	2.53 (2.47–2.59)	221,551	79.31 (78.97–79.65)
**2012**	13,875	4.24 (4.17–4.31)	6,990	2.20 (2.15–2.25)	213,965	74.58 (74.25–74.91)
**2013**	15,095	4.52 (4.45–4.60)	7,370	2.25 (2.20–2.31)	217,625	74.53 (74.20–74.85)
**2014**	16,340	4.81 (4.73–4.89)	7,395	2.21 (2.16–2.27)	223,525	75.11 (74.79–75.44)
**2015**	21,385	6.16	11,320	2.83	224,305	73.93

© 2026 Martin BI et al. *JAMA Network Open*.

		(6.07 - 6.24)		(2.77 – 2.89)				(73.62 - 74.25)		

	AP 1-level		AP 2-7 level		Complex		1-column 1-level		1-column 2-7 level	
	Volume	Rate (95%CI)	Volume	Rate (95%CI)	Volume	Rate (95%CI)	Volume	Rate (95%CI)	Volume	Rate (95%CI)
**2016**	29,835	8.58 (8.47–8.68)	25,860	6.37 (6.29-6.46)	34,315	9.69 (9.58–9.80)	106,505	33.32 (33.09–33.54)	84,955	28.05 (27.85–28.25)
**2017**	34,605	9.78 (9.67–9.89)	28,400	6.86 (6.77–6.95)	39,720	10.96 (10.84–11.07)	99,110	30.46 (30.25–30.67)	79,335	25.64 (25.45–25.83)
**2018**	50,640	14.13 (13.99–14.27)	39,665	9.42 (9.31–9.53)	46,350	12.53 (12.40–12.65)	82,440	24.99 (24.78–25.17)	65,085	20.67 (20.50–20.83)
**2019**	58,895	16.23 (16.08–16.39)	45,555	10.64 (10.52–10.76)	49,900	13.22 (13.09–13.35)	73,600	22.01 (21.84–22.18)	60,640	18.93 (18.77–19.09)
**2020**	50,810	13.83 (13.69–13.97)	43,775	10.07 (9.95–10.18)	46,335	12.05 (11.93–12.18)	58,785	17.34 (17.19–17.50)	52,885	16.23 (16.09–16.38)
**2021**	49,915	13.40 (13.27–13.54)	44,490	10.07 (9.96–10.19)	47,410	12.19 (12.07–12.31)	53,275	15.53 (15.38–15.67)	53,505	16.23 (16.09–16.38)
**2022**	51,675	13.77 (13.63–13.90)	47,250	10.59 (10.47–10.71)	50,525	12.79 (12.66–12.91)	52,925	15.27 (15.13–15.41)	54,220	16.21 (16.06–16.35)
**2023**	57,955	15.26 (15.12–15.41)	54,215	11.98 (11.86–12.11)	54,620	13.61 (13.48–13.74)	51,175	14.58 (14.44–14.71)	55,270	16.29 (16.15–16.43)

© 2026 Martin BI et al. *JAMA Network Open*.

eFigure. Age-Specific Rate (per 100 000) and Volume (in Thousands) of Inpatient Lumbar Fusion in the United States for 2002 and 2023

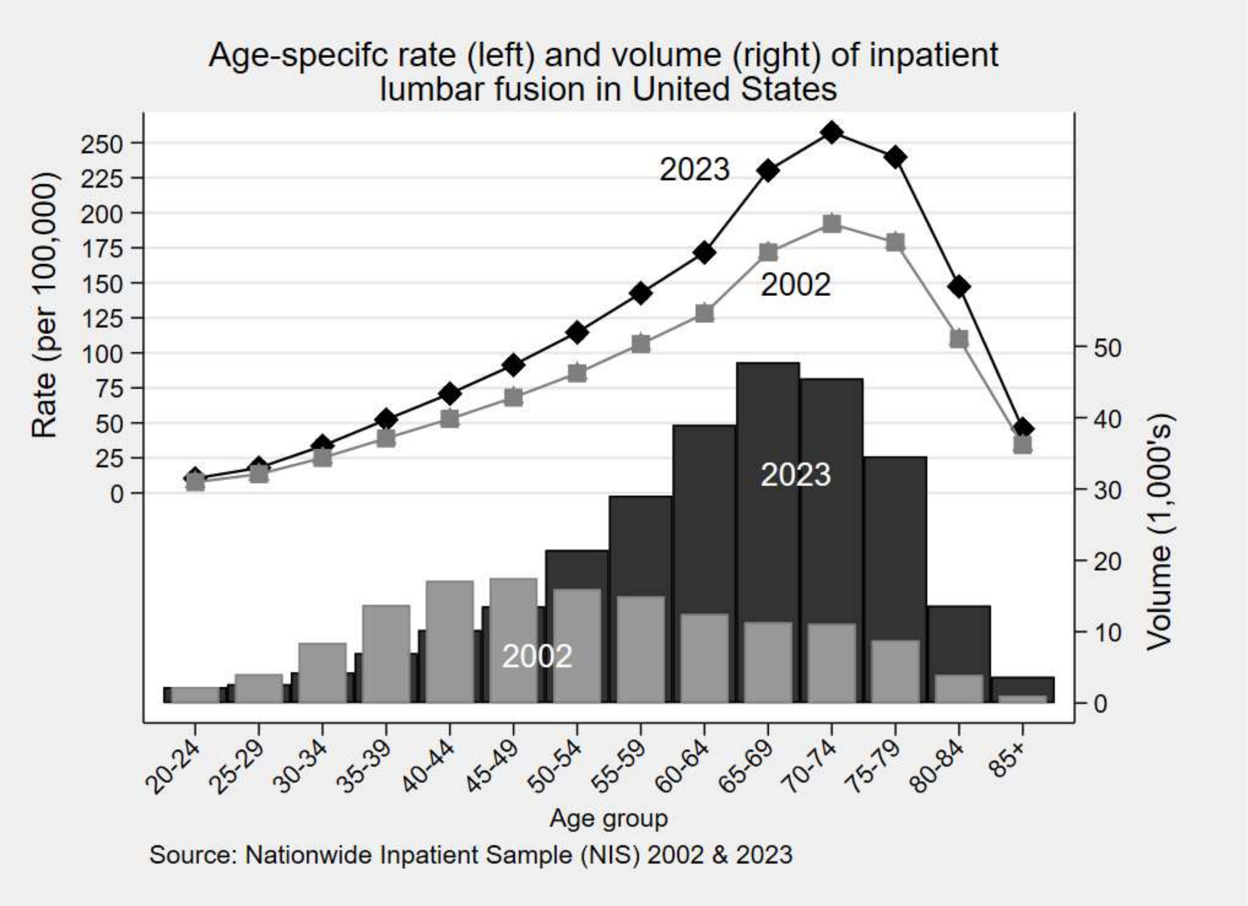

© 2026 Martin BI et al. *JAMA Network Open*.
