# Supplementary material for: Cost and Utilization Trends of Lumbar Fusion
Source: JAMA Netw Open. 2026 Mar 4;9(3):e260452. doi: 10.1001/jamanetworkopen.2026.0452 (PMC12961518; doi:10.1001/jamanetworkopen.2026.0452)
Supplement: Supplement 2. — Data Sharing Statement [file jamanetwopen-e260452-s002.pdf]

Data Sharing Statement

Martin. Cost and Utilization Trends of Lumbar Fusion. *JAMA Netw Open*. Published March 04, 2026. doi:10.1001/jamanetworkopen.2026.0452

Data
Data available: No

Additional Information
Explanation for why data not available: The data for this study come from the National Inpatient Sample (NIS) and the Nationwide Ambulatory Surgery Sample (NASS), publicly available databased from the Agency for Healthcare Research and Quality's Healthcare Cost and Utilization Project (HCUP). The raw NIS and NASS data files can purchased from HCUP after signing a Data Use Agreement. We will not share the individual patient-level data extracted from the NIS or NASS due to restrictions imposed by the HCUP Data Use Agreement, which prohibits the direct distribution of the data files. However, the complete statistical/analytical code (e.g., Stata scripts) and data dictionary defining the variables used the analysis will be made available upon request to the corresponding author. The supplemental material included the coding algorithms we used for defining procedural case mix and indication.
